# Recurrent hemorrhage risk associated with medial target medullary artery anastomosis from the periventricular collateral vessel in adult patients with moyamoya disease

**DOI:** 10.1186/s12883-021-02130-x

**Published:** 2021-03-06

**Authors:** Jian Wang, Qingrong Zhang, Xia Lu, Qun Liang, Yi Wang, Yichao Zhu, Shijie Na, Fang Liu, Liming Tang, Yongbo Yang

**Affiliations:** 1 Department of Neurosurgery, The Affiliated Changzhou No. 2 People’s Hospital of Nanjing Medical University, Changzhou, China; 2 Comprehensive stroke center, The Affiliated Changzhou No. 2 People’s Hospital of Nanjing Medical University, Changzhou, China; 3 Department of Neurosurgery, The Affiliated Drum Tower Hospital of Nanjing University Medical School, Nanjing, China; 4 Department of Neurosurgery, Xuanwu Hospital Capital Medical University, Beijing, China; 5 Department of Physiology, Nanjing Medical University, Nanjing, China; 6 Department of Gastrointestinal Surgery, The Affiliated Changzhou No. 2 People’s Hospital of Nanjing Medical University, Changzhou, China

**Keywords:** Moyamoya disease, Intracranial hemorrhage, Target anastomotic territory, Natural history, Angiography

## Abstract

**Background:**

Although the association between periventricular target collateral anastomosis and recurrent ipsilateral hemorrhage has been evaluated in adult patients with moyamoya disease (MMD), no studies have investigated the relationship between target anastomotic territory and recurrent ipsilateral hemorrhage. The goal of this study was to assess this association.

**Methods:**

Consecutive adult MMD patients who had experienced initial intracranial hemorrhage and undergone conservative treatment were included. Two readers assessed angiographic results to identify the target anastomotic territory (medial medullary artery, lateral medullary artery, multiple medullary arteries, or nonmedullary artery) responsible for the hemorrhage. Cox proportional hazard regression models were used to estimate the risk of recurrent hemorrhage.

**Results:**

In the 36 hemispheres with initial hemorrhage, the target anastomotic territory was in the anastomotic territory of the medial medullary artery in 10 (27.8%), lateral medullary artery in 15 (41.7%), multiple medullary arteries in 2 (5.6%), and a nonmedullary artery in 9 (25.0%) hemispheres. During 45.1 ± 40.0 months of follow-up, recurrent ipsilateral hemorrhage occurred in 44.4% (16/36) of hemispheres. The target anastomotic territories responsible for the recurrent event were in the anastomotic territory of the medial medullary artery in 9 (56.3%) hemispheres, lateral medullary artery in 6 (37.5%) hemispheres, and multiple medullary arteries in 1 (6.3%) hemisphere. The anastomotic territory of the medial medullary artery was associated with recurrent hemorrhage before (HR = 2.94; 95% CI, 1.07–8.08; *p* = 0.037) and after (HR = 6.65; 95% CI, 1.32–33.60; *p* = 0.022) adjustments were made for confounding factors.

**Conclusions:**

The incidence of recurrent ipsilateral hemorrhage varies with the target anastomotic territory in adult patients with MMD. Medial target medullary artery anastomosis is a significant risk factor for recurrent ipsilateral hemorrhage.

## Background

Moyamoya disease (MMD) is a cerebrovascular disorder that is characterized by progressive occlusion in the terminal portion of the internal carotid artery (ICA) and in its main branches within the circle of Willis [1]. Intracranial hemorrhage accounts for half of the primary manifestations of MMD in adult patients, and the hemorrhage recurrence rate is as high as 44% during the natural course of the disease [2–5]. Prevention of recurrent hemorrhage is therefore pivotal in terms of disease management.

Surgical revascularization to reduce the incidence of recurrent hemorrhage is the only effective treatment for patients with hemorrhagic MMD [2, 6, 7]. The goal of revascularization surgery is to prevent recurrent hemorrhage by eliminating persistent hemodynamic stress from the anastomotic territory in the abnormally extended target collateral vessels. Thus, the risk of recurrent hemorrhage in various target territories must be evaluated. Previous studies have identified several risk factors related to recurrent hemorrhage including the hemorrhagic type [5], the hemorrhagic site [8], and the target collateral vessels involved [9, 10]. However, the characteristics of the target anastomotic territory responsible for hemorrhage and the relationship between this territory and recurrent ipsilateral hemorrhage are still unclear.

In this retrospective study, we therefore sought to assess the association of the target anastomotic territory with the risk of recurrent ipsilateral hemorrhage in the natural history of adult patients with MMD.

## Methods

### Study population and design

This study, which is a follow-up to previous research [4], was approved by our institutional review board with a waiver of informed consent. The primary design of the study has been described elsewhere [4]. In brief, consecutive adult patients with angiographically demonstrated MMD (diagnosed according to the guidelines proposed by the Ministry of Health, Labour and Welfare of Japan) [11] who had experienced initial intracranial hemorrhage, received conservative treatment, and had > 5 years of clinical follow-up at a single institution between January 2008 and December 2018 were recruited. Patients with factors associated with non-MMD-related hemorrhage and with factors influencing evaluation of the target collateral vessel were excluded from the study. Additional details regarding the inclusion and exclusion criteria have been described previously [4].

After baseline investigation, all patients underwent conservative treatment, which included control of hypertension, diabetes mellitus, and atherosclerotic plaque. The use of antiplatelet drugs was not permitted unless the patient was experiencing cerebral ischemia. Outpatient clinical follow-up was performed every 3 to 6 months. The occurrence of recurrent hemorrhage was recorded if there was an acute neurologic symptom during follow-up with a corresponding new intracranial hemorrhage evident on brain imaging. Demographic and clinical characteristics of study patients were collected from the medical records.

### Variables of interest

Identifying the target anastomotic territory for the hemorrhage, the primary variable of interest, was a systematic process consisting of the following 3 steps: 1) identification of the hemorrhagic site associated with the present hemorrhagic event; 2) identification of the target collateral vessels responsible for the hemorrhage; and 3) identification of the target anastomotic territory fed by the target collateral vessels. Assessment of imaging scans for the target collateral vessel was performed via the baseline angiography through consensus reading by 2 raters, each with 5 years’ experience in MMD imaging evaluation, who were blinded to treatment and clinical outcome. In cases of disagreement between the 2 raters, a third investigator with > 10 years’ experience in MMD imaging resolved any discrepancies between the 2 raters. Both raters had participated in a training session involving 15 representative cases (including 5 recurrent cases) in which the target collateral vessels had been confirmed by angiography.

**Fig. 1:**
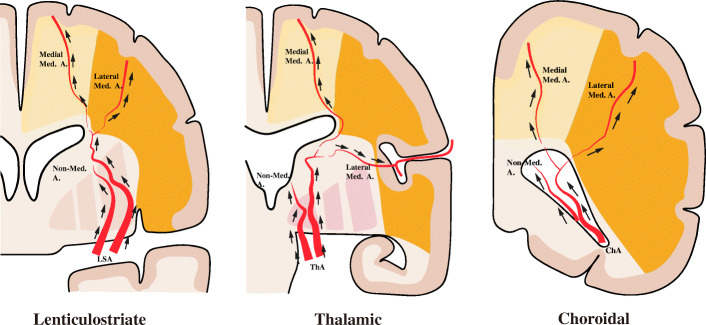
Schematic illustrations demonstrating imaging definitions of the medial, lateral, and nonmedullary artery anastomotic territories in different target collateral vessel patterns. Med = medullary; A = artery; LSA = lenticulostriate artery; ThA = thalamic artery; ChA = choroidal artery

We defined the target anastomotic territory as the feeding territory from the terminal portion of the target collateral vessel, located beyond (with collateral anastomosis) or at (without collateral anastomosis) the hemorrhagic site, as seen on frontal and lateral views in the later arterial phase of angiography. The target anastomotic territory was thus classified as the anastomotic territory of the medial medullary artery, lateral medullary artery, multiple medullary arteries, or a nonmedullary artery based on the anatomical and radiological evidence [12, 13]; medullary arteries were defined as arteries supplying the cerebral white matter. The anastomotic territory of the medial medullary artery was defined as the area fed by the cortical branches of the anterior cerebral artery (ACA) and the medial branches of the posterior cerebral artery (PCA) (parietooccipital artery). The anastomotic territory of the lateral medullary artery was defined as the area fed by the cortical branches of the middle cerebral artery (MCA). The anastomotic territory for multiple medullary arteries was defined as the area fed by the cortical branches of any two of the above-mentioned major arteries (ACA, MCA, and PCA). The anastomotic territory of the nonmedullary artery was defined as the area located at the basal ganglia region and thalamus and fed by the basal perforators (medial and lateral striate arteries, thalamic perforators, and choroidal arteries). Schematic illustrations of these definitions are shown in Fig. 1.

Previously reported angiographic definitions of the periventricular collateral vessels, which derive from the lenticulostriate artery (LSA), thalamic artery (ThA), and choroidal artery (ChA), were adapted to the present study [10]. The target collateral vessel was defined as an abnormally extended periventricular collateral vessel corresponding to the distribution of the presumed hemorrhagic site on angiography. The hemorrhagic site was generally identified with noncontrast-enhanced CT performed during the acute phase of the hemorrhage. When the hemorrhage presented as diffuse ventricular hemorrhage, susceptibility-weighted imaging (SWI) was performed within 2 months of the hemorrhagic event to identify the presumed hemorrhage origin, which was defined as an abnormal hypointense area > 10 mm that overlapped with the signal of the abnormally extended collateral vessel on SWI and was also apparent on T2-weighted imaging. The initial presumed hemorrhagic site was considered to be responsible for the recurrent hemorrhage when the recurrent hemorrhage occurred in the same hemisphere containing the initial target collateral vessel and corresponded to the distribution of recurrent hemorrhagic sites.

Other baseline variables were also evaluated, such as the occurrence of posterior hemorrhage [8], the presence of ruptured collateral aneurysm, the Suzuki stage of the disease [1], and involvement of the PCA (Mugikura stages II-IV) [14].

### Statistical analysis

All statistical analyses were performed using SPSS 22.0 (IBM). Continuous variables were described as mean ± SD, and categorical variables were presented as number and percentage. A Mann-Whitney U test and a chi-square or Fisher exact test were used to compare hemispheres with and without recurrent ipsilateral hemorrhage in terms of baseline angiographic characteristics. Univariate and multivariate Cox proportional hazard regression models were used to estimate the risk of recurrent ipsilateral hemorrhage for the target anastomotic territories. A *p* value < 0.05 was considered significant.

## Results

**Fig. 2:**
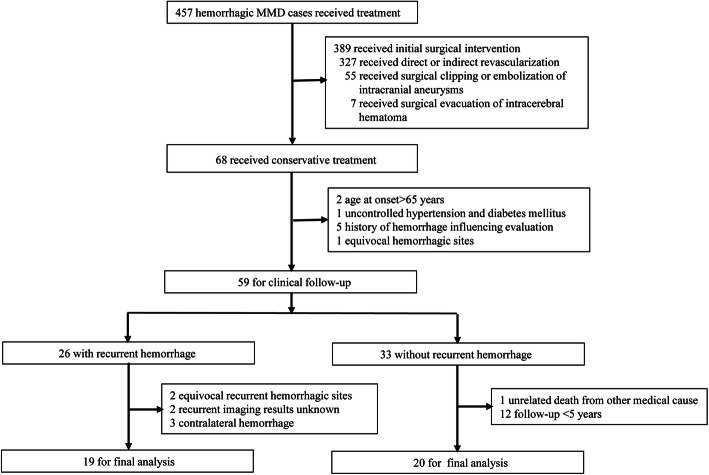
Flowchart of patient selection

From January 2008 to December 2018, a total of 457 patients with MMD presented with intracranial hemorrhage and were treated at a single tertiary care medical center; 68 (14.9%) of these underwent conservative treatment. The reasons for using conservative treatment in these patients were as follows: adequate external carotid artery compensation (*n* = 9); Modified Rankin Scale (mRS) score > 3 (*n* = 27); presence of subarachnoid hemorrhage without target intracranial aneurysm at baseline (*n* = 2); patient choice based on economic reasons (*n* = 21); and recurrent hemorrhage occurring during the waiting period for revascularization (*n* = 9). No significant differences were observed in baseline demographic factors between patients with and those without surgical intervention (Table 1). Of these 68 patients, 29 with non-MMD-related hemorrhage and factors influencing evaluation of the target collateral vessel were excluded at baseline or during the follow-up period. Thus, a total of 39 patients met the initial eligibility criteria for inclusion in the study (Fig. 2). Of these, 3 patients with ruptured intracranial aneurysms were excluded because the target collateral vessels (parent artery) originated from major branches of the PCA (*n* = 2) or from the meningeal middle artery (*n* = 1). The remaining 36 patients (27 women; mean age at diagnosis, 44.9 ± 10.1 years) were included in the final analysis (Table 2).

**Table 1:** Baseline demographic characteristics for hemorrhagic MMD patients with and without surgery

Characteristic	Patients Without Surgery (*n* = 68)	Patients With Surgery (*n* = 389)	*p* Value
Women	43 (63.2)	260 (66.8)	0.562
Mean age ± SD, yrs	45.0 ± 8.1	45.4 ± 7.4	0.699
Smokers	8 (11.8)	35 (9.0)	0.471
Concurrent disease			
Hypertension	24 (35.3)	128 (32.9)	0.700
Dyslipidemia	12 (17.6)	77 (19.8)	0.680
Diabetes mellitus	9 (13.2)	78 (20.1)	0.187
History of stroke	8 (11.8)	35 (9.0)	0.471

*MMD* moyamoya disease

Values are numbers of patients (%) unless otherwise indicated

**Table 2:** Baseline characteristics of study patients (*N* = 36)

Characteristic	Value
Women	27 (75.0)
Mean age ± SD, yrs	44.9 ± 10.1
Smokers	2 (5.6)
Concurrent disease	
Hypertension	13 (36.1)
Dyslipidemia	8 (22.2)
Diabetes mellitus	3 (8.3)
History of ischemia	3 (8.3)
Hemorrhagic type	
IVH	16 (44.4)
ICH + IVH	10 (27.8)
ICH	9 (25.0)
ICH + SAH	1 (2.8)
Hemorrhagic site	
Thalamus	3 (8.3)
Periventricular	23 (63.9)
Basal ganglia	7 (19.4)
Corpus callosum	3 (8.3)
Presence of posterior hemorrhage	21 (58.3)
Presence of ruptured collateral aneurysm	2 (5.6)
Suzuki stage	
I-II	1 (2.8)
III-IV	27 (75.0)
V-VI	8 (22.2)
PCA involved	9 (25.0)

*ICH* intracerebral hemorrhage, *IVH* intraventricular hemorrhage, *SAH* subarachnoid hemorrhage, *PCA* posterior cerebral artery

Values are numbers of patients (%) unless otherwise indicated

### Baseline variable analysis

In 36 initial hemorrhagic hemispheres, the hemorrhagic sites were in the periventricular area in 23 hemispheres (63.9%), the basal ganglia in 7 hemispheres (19.4%), the corpus callosum in 3 hemispheres (8.3%), and the thalamus in 3 hemispheres (8.3%). Of the 36 hemispheres, the target collateral vessel derived from the ChA in 25 (69.4%), the LSA in 8 (22.2%), and the ThA in 3 (8.3%) hemispheres.

Among the target anastomotic territories, 10 (27.8%) were classified as the anastomotic territory of the medial medullary artery, including the posterior internal frontal artery (*n* = 1), paracentral artery (*n* = 6), superior internal parietal artery (*n* = 2), and parietooccipital artery (*n* = 1); 15 (41.7%), as the anastomotic territory of the lateral medullary artery, including the central artery (*n* = 8), precentral artery (*n* = 2), anterior parietal artery (*n* = 1), and angular gyrus artery (*n* = 4); 2 (5.6%), as the anastomotic territory of multiple medullary arteries, including the anterior parietal artery, paracentral artery, and angular gyrus artery (*n* = 1) and the paracentral artery and central artery (*n* = 1); and 9 (25.0%), as the anastomotic territory of a nonmedullary artery.

Among the target anastomotic territories, the ChA most commonly fed the anastomotic territory of multiple medullary arteries (2/2; 100.0%), followed by the medial medullary artery (9/10; 90.0%), lateral medullary artery (10/15; 66.7%), and nonmedullary artery (4/9; 44.4%). LSA most commonly fed the anastomotic territory of the nonmedullary artery (4/9; 44.4%), followed by the lateral medullary artery (4/15; 26.7%). None fed the anastomotic territory of multiple medullary arteries or the medial medullary artery. ThA involvement was distributed evenly among the three anastomotic subgroups (with the exception of multiple medullary arteries).

Advanced Suzuki disease stages (Suzuki stage > III) were most commonly associated with the target anastomotic territory of the medial medullary artery (5/10; 50.0%), followed by the anastomotic territories of the nonmedullary artery (2/9; 22.2%) and the lateral medullary artery (3/15; 20.0%). Involvement of the PCA was most common in the anastomotic territory of the medial medullary artery (4/10; 40.0%), followed by the anastomotic territories of the lateral medullary artery (4/15; 26.7%) and the nonmedullary artery (1/9; 11.1%).

### Recurrent hemorrhage

During the mean 45.1 ± 40.0 months (range, 1–114 months) of follow-up, 16 of the 36 patients (44.4%) experienced recurrent ipsilateral hemorrhage. In these 16 cases, the recurrent hemorrhagic sites were in the periventricular area in 11 hemispheres (68.8%), the basal ganglia in 3 hemispheres (18.8%), and the corpus callosum in 2 hemispheres (12.5%). The target collateral vessel derived from the ChA in 12 cases (75.0%), the LSA in 3 cases (18.7%), and the ThA in 1 case (6.3%). Of the target anastomotic territories responsible for the recurrent hemorrhage, 9 (56.3%) were classified as the anastomotic territory of the medial medullary artery; 6 (37.5%), as the anastomotic territory of the lateral medullary artery; and 1 (6.3%), as the anastomotic territory of multiple medullary arteries. No recurrent hemorrhages were located at the anastomotic territory of a nonmedullary artery. Eleven (6 women; age range at diagnosis, 36–63 years) recurrent hemorrhages were located in the territory involved in the initial hemorrhage (Table 3), and 5 (3 women; age range at diagnosis, 42–51 years) were located in a different target anastomotic territory (Table 4).

**Table 3:** Clinical features of 11 recurrent ipsilateral hemorrhages in the same target anastomotic territory involved in the initial hemorrhage

Case	Hemorrhagic Type	Target Anastomotic Territory	Target Vessel	Interval (mo)
1	IVH	Posterior internal frontal artery (medial)	ChA	5
2	IVH	Paracentral artery (medial)	ChA	10
3	IVH	Paracentral artery (medial) and central artery (lateral)	ChA	2
4	IVH	Superior internal parietal artery (medial)	ChA	10
5	ICH + IVH	Paracentral artery (medial)	ChA	49
6	IVH	Superior internal parietal artery (medial)	ChA	1
7	IVH	Paracentral artery (medial)	ChA	5
8	ICH	Central artery (lateral)	ChA	47
9	ICH + IVH	Paracentral artery (medial)	ChA	14
10	IVH	Central artery (lateral)	LSA	59
11	ICH + SAH	Central artery (lateral)	LSA	1

*ICH* intracerebral hemorrhage, *IVH* intraventricular hemorrhage, *SAH* subarachnoid hemorrhage, *ChA* choroidal artery, *LSA* lenticulostriate artery

**Table 4:** Clinical features of 5 recurrent ipsilateral hemorrhages in a target anastomotic territory different from that involved in the initial hemorrhage

Case	Hemorrhagic Type	Target Anastomotic Territory (Initial/Recurrent)	Target Vessel (Initial/Recurrent)	Interval (mo)
1	IVH	Central artery (lateral)/ Paracentral artery (medial)	ChA/ ChA	4
2	IVH	Precentral artery (lateral)/ Paracentral artery (medial)	ChA/ ChA	12
3	IVH	Deep white matter/ Central artery (lateral)	ChA/ ChA	58
4	ICH + IVH	Anterior parietal artery (lateral)/ Angular gyrus artery (lateral)	ChA/ ChA	1
5	ICH + IVH	Angular gyrus artery (lateral)/ Central artery (lateral)	ThA/ LSA	25

*ICH* intracerebral hemorrhage, *IVH* intraventricular hemorrhage, *ChA* choroidal artery, *LSA* lenticulostriate artery, *ThA* thalamic artery

### Association between target anastomotic territory subgroup and recurrent Ipsilateral hemorrhage

**Fig. 3:**
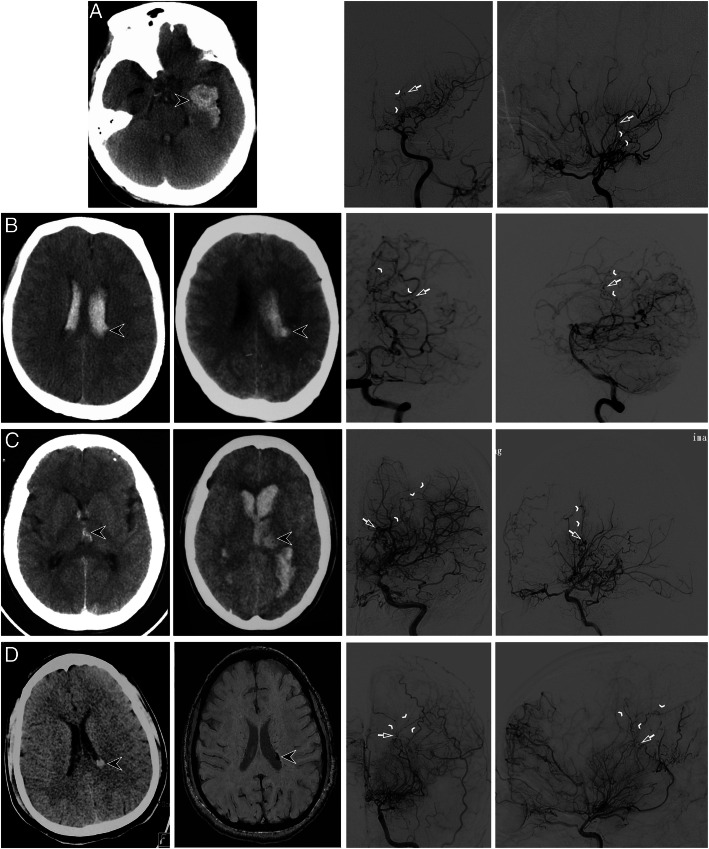
Representative cases of target anastomotic territory patterns. Hemorrhagic sites during the acute phase are indicated by black arrowheads on noncontrast-enhanced CT scans. Target collateral vessels are indicated by white arrowheads and hemorrhagic sites are indicated by white arrows on angiograms. **a:** A 58-year-old man experienced an intraventricular hemorrhage and was treated with conservative therapy. A CT image *(left)* indicates the initial hemorrhagic site located at the left temporal horn of the lateral ventricle. Left anterior-posterior *(middle)* and lateral *(right)* carotid artery angiograms obtained at baseline demonstrate the anterior choroidal artery responsible for the hemorrhage. No obvious medullary artery anastomosis could be observed. After > 70 months of clinical follow-up, no recurrent hemorrhage had occurred. **b:** A 50-year-old man experienced a recurrent hemorrhage in the ipsilateral hemisphere. A CT image *(left)* indicates the initial hemorrhage in the posterior portion of the body of the left lateral ventricle. A CT image obtained 49 months later *(second from left)* demonstrates a recurrent hemorrhage in the initial hemorrhagic site. Anterior-posterior (*second from right*) and lateral (*right*) views of the left vertebral angiogram obtained at baseline reveal the lateral posterior choroidal artery responsible for the initial and recurrent hemorrhages. The angiograms demonstrate obvious medial medullary artery anastomosis between the target collateral vessel and the territory fed by the paracentral artery. (Modified with permission from Wang et al. *AJNR Am J Neuroradiol*. 2019;40(10):1665–1671.^4^). **c:** A 36-year-old woman experienced a recurrent hemorrhage in the ipsilateral hemisphere. A CT image *(left)* demonstrates the initial hemorrhage in the body of the left lateral ventricle. A CT image obtained 49 months later *(second from left)* demonstrates a recurrent hemorrhage near the initial hemorrhagic site. Anterior-posterior (*second from right*) and lateral (*right*) views of the left carotid angiogram obtained at baseline reveal the lenticulostriate artery anastomosis responsible for the initial and recurrent hemorrhages. The angiograms demonstrate obvious lateral medullary artery anastomosis between the target collateral vessel and the central artery territory. **d:** A 36-year-old man experienced an intraventricular hemorrhage and was treated with conservative therapy. CT images (confirmed by susceptibility-weighted imaging) *(left and second from left)* indicate the initial hemorrhagic site located at the left posterior body of the lateral ventricle. Left anterior-posterior *(second from right)* and lateral *(right)* carotid artery angiograms obtained at baseline demonstrate the anterior choroidal artery responsible for the hemorrhage. The angiograms demonstrate obvious multiple medullary artery anastomosis between the target collateral vessel and the anterior parietal artery, paracentral artery, and angular gyrus artery territory. After > 96 months of clinical follow-up, no recurrent hemorrhage had occurred

Representative cases demonstrating the various target anastomotic territory subgroups are shown in Fig. 3. Hemispheres with recurrent ipsilateral hemorrhage had a significantly lower prevalence (6.3% [1/16] vs 40.0% [8/20]; *p* = 0.026) of the target anastomotic territory of the nonmedullary artery and a slightly higher prevalence (43.8% [7/16] vs 15.0% [3/20]; *p* = 0.073) of the target anastomotic territory of the medial medullary artery when compared with hemispheres without recurrent ipsilateral hemorrhage; this difference was not observed for hemispheres with hemorrhage in the target anastomotic territory of the lateral medullary artery (43.8% [7/16] vs 40.0% [8/20]; *p* > 0.99) or multiple medullary arteries (6.3% [1/16] vs 5.0% [1/20]; *p* > 0.99).

Univariate Cox regression analysis demonstrated a significant association between the target anastomotic territory of the medial medullary artery and recurrent ipsilateral hemorrhage (HR = 2.94; 95% CI, 1.07–8.08; *p* = 0.037). In multivariate Cox regression analysis, after adjustments were made for age, sex, presence of posterior hemorrhage, target collateral vessel derived from the ChA, advanced Suzuki stage, involvement of the PCA, and presence of ruptured collateral aneurysm, this association remained significant (HR = 6.65; 95% CI, 1.32–33.60; *p* = 0.022; Table 5).

**Table 5:** Association of recurrent ipsilateral hemorrhage with clinical and vascular characteristics

	Risk of Recurrent Hemorrhage					
	Univariate Analysis			Multivariate Analysis		
	HR	95% CI	*p* Value	HR	95% CI	*p* Value
Sex, female	1.01	0.33–3.14	0.985	–	–	–
Age	1.03	0.97–1.08	0.371	–	–	–
Smokers	1.28	0.17–9.71	0.812	–	–	–
Hypertension	0.79	0.27–2.28	0.662	–	–	–
Dyslipidemia	1.05	0.34–3.26	0.932	–	–	–
Diabetes mellitus	0.62	0.08–4.68	0.640	–	–	–
Advanced Suzuki stage	1.29	0.45–3.72	0.636	–	–	–
Involvement of PCA	1.43	0.50–4.13	0.507	–	–	–
Presence of posterior hemorrhage	1.27	0.46–3.49	0.646	–	–	–
Presence of ruptured collateral aneurysm	3.30	0.75–14.58	0.115	–	–	–
Target collateral vessel derived from ChA	1.51	0.49–4.68	0.477	–	–	–
Target anastomotic territory						
Nonmedullary artery anastomosis	0.14	0.02–1.04	0.054	0.11	0.01–1.03	0.053
Lateral medullary artery anastomosis	1.09	0.41–2.94	0.858	0.56	0.15–2.13	0.397
Medial medullary artery anastomosis	2.94	1.07–8.08	0.037	6.65	1.32–33.60	0.022
Multiple medullary artery anastomosis	1.47	0.19–11.12	0.712	2.17	0.22–21.66	0.509

*PCA* posterior cerebral artery, *ChA* choroidal artery

Multivariate analysis was adjusted for confounding factors including initial age, sex, presence of posterior hemorrhage, target collateral vessel derived from the choroidal artery, advanced Suzuki stage, involvement of the posterior cerebral artery, and presence of ruptured collateral aneurysm

## Discussion

This study demonstrated that the target anastomotic territory of the medial medullary artery is significantly associated with recurrent ipsilateral hemorrhage in adult patients with MMD. These findings suggest that imaging evaluations and surgical interventions for hemorrhagic MMD should focus not merely on the hemorrhagic sites and periventricular collateral anastomosis but also on the target anastomotic territory.

Despite limited data from randomized clinical trials, the current recommendation is to perform direct surgical revascularization in patients with hemorrhagic MMD because of the dismal prognosis of this disease subtype. The Japan Adult Moyamoya (JAM) Trial demonstrated that the effectiveness of direct surgical bypass in reducing recurrent hemorrhage during 5 years of follow-up is moderate [2]. A detailed pretreatment evaluation of risk factors associated with recurrent hemorrhage has therefore been recognized as critical. Previous studies have identified several such risk factors, including the occurrence of periventricular hemorrhage [5], particularly in the posterior [8], and the presence of choroidal collateral anastomosis [9, 10]. However, most of these studies did not address target anastomotic territory as a potential risk factor.

In this study, we found that hemispheres with a target anastomotic territory of the medial medullary artery, which is fed mainly by choroidal collateral vessels, demonstrated an increased risk of recurrent ipsilateral hemorrhage. These results support findings from previous studies demonstrating that choroidal collateral anastomosis is a predictor of recurrent hemorrhage in MMD [4, 9]. However, the mechanism underlying this association is not fully understood. Here, we propose a possible explanation for this relationship.

In this study, we found that the target anastomotic territory of the medial medullary artery in hemispheres with recurrent hemorrhage was mostly distributed near the central areas fed by the ACA branches, including the posterior internal frontal artery, paracentral artery, and superior internal parietal artery. According to the innate compensation for the target territory, the pathway from the branches of the PCA (posterior pericallosal artery) may be a prominent route to the above territory. However, we found that stenotic-occlusive lesions involved one-third of PCAs in the anastomotic hemispheres of the medial medullary artery, a proportion higher than was seen in the other target anastomotic territories. In addition, the hemispheres with target anastomotic territory of the medial medullary artery had a higher prevalence of disease classified as an advanced Suzuki stage. Hemodynamic decompensation is thus added to the medial central areas, a watershed between the ACA and PCA. Further research is needed to verify this hypothesis.

Because the target anastomotic territory of the medial medullary artery appears to be an independent predictor of recurrent ipsilateral hemorrhage, targeted surgical treatment is warranted in these cases. The ACA territory is an important area of intellectual and cognitive functioning; chronic hypoperfusion of this territory has been associated with decreases in cognitive and intellectual ability [15, 16]. However, revascularization of this area has not drawn as much attention as revascularization of the MCA territory. Previous studies have reported treating ACA hypoperfusion with indirect revascularization methods such as encephalogaleosynangiosis [17], encephaloduroperiostealsynangiosis [18], or burr-hole surgery [19]; however, the effectiveness of these methods for eliminating persistent hemodynamic stress from the target anastomotic territory remains unclear. In a recent pilot study, a tailored targeting bypass strategy using an occipital artery-to-ACA bypass to prevent hemorrhage was found to allow for target anastomotic territory intervention in this population [20]. A large case series is needed to determine the potential value of this intervention in preventing recurrent hemorrhage in hemispheres with a target anastomotic territory of the medial medullary artery.

This analysis has several limitations, which are similar to those reported in our previous study [4]. First, the number of patients was relatively small because of the rarity of nonsurgical intervention in patients with hemorrhagic MMD, and we excluded nearly 50% of cases, which could have led to selection bias. Second, the relationship between target anastomotic territory and recurrent hemorrhage was determined only via analysis of the initial angiographic images; follow-up imaging validation of the target collateral vessels was lacking. Third, the subgroup analysis of target anastomotic territories was ambiguous (single or multiple target anastomotic territory) and was limited by the use of angiographic images for final evaluation in 1 case. In a recent study, SWI + time of flight (TOF) magnetic resonance angiography images were found to provide information regarding periventricular collateral anastomosis [21], suggesting that magnetic resonance imaging may be more accurate than angiography for target anastomotic territory assessment. Another recent study demonstrated that hemodynamic factors may play a role in subsequent hemorrhagic stroke risk among patients with MMD; however, larger studies are needed to test this hypothesis in patients with recurrent hemorrhage [22]. Despite these limitations, the current study remains one of the first to analyze the association between target anastomotic territory and recurrent ipsilateral hemorrhage in patients with MMD. Future studies are warranted to assess the use of surgical intervention in the medial anastomotic territory fed by the target periventricular collateral vessels, and long-term follow-up is also needed to determine the efficacy of this treatment in preventing recurrent hemorrhage.

## Conclusion

This study demonstrated that the incidence of recurrent ipsilateral hemorrhage varies depending on the initial target anastomotic territory responsible for hemorrhage. Specifically, we found that hemorrhagic hemispheres with a target anastomotic territory of the medial medullary artery are at higher risk for recurrent ipsilateral hemorrhage. These findings support a possible underlying mechanism for recurrent hemorrhage that may allow for the development of novel strategies to prevent recurrent hemorrhage in adult patients with MMD. However, further studies in larger cohorts are needed.

## Data Availability

The datasets that were collected and/or analyzed during the current study are available from the corresponding author upon reasonable request.

## Abbreviations

MMD: Moyamoya disease
DSA: Digital subtraction angiography;
ICA: Internal carotid artery
ACA: Anterior cerebral artery
MCA: Middle cerebral artery
PCA: Posterior cerebral artery
ChA: Choroidal artery
LSA: Lenticulostriate artery
ThA: Thalamic artery
SWI: Susceptibility-weighted imaging
JAM Trial: Japan Adult Moyamoya Trial
TOF: Time of flight
HR: Hazards ratio
CI: Confidence interval
SD: Standard deviation
